# Characterizing the Structural Pattern Predicting Medication Response in Herpes Zoster Patients Using Multivoxel Pattern Analysis

**DOI:** 10.3389/fnins.2019.00534

**Published:** 2019-05-28

**Authors:** Ping Zeng, Jiabin Huang, Songxiong Wu, Chengrui Qian, Fuyong Chen, Wuping Sun, Wei Tao, Yuliang Liao, Jianing Zhang, Zefan Yang, Shaonan Zhong, Zhiguo Zhang, Lizu Xiao, Bingsheng Huang

**Affiliations:** ^1^School of Biomedical Engineering, Health Science Center, Shenzhen University, Shenzhen, China; ^2^Clinical Research Center for Neurological Diseases, Shenzhen University, Shenzhen, China; ^3^Department of Pain Medicine and Shenzhen Municipal Key Laboratory for Pain Medicine, Shenzhen Sixth Hospital of Guangdong Medical University, Shenzhen, China; ^4^Department of Neurosurgery, Shenzhen University General Hospital, Shenzhen, China

**Keywords:** herpes zoster, medication response, structural MRI, multivoxel pattern analysis, prediction

## Abstract

Herpes zoster (HZ) can cause a blistering skin rash with severe neuropathic pain. Pharmacotherapy is the most common treatment for HZ patients. However, most patients are usually the elderly or those that are immunocompromised, and thus often suffer from side effects or easily get intractable post-herpetic neuralgia (PHN) if medication fails. It is challenging for clinicians to tailor treatment to patients, due to the lack of prognosis information on the neurological pathogenesis that underlies HZ. In the current study, we aimed at characterizing the brain structural pattern of HZ before treatment with medication that could help predict medication responses. High-resolution structural magnetic resonance imaging (MRI) scans of 14 right-handed HZ patients (aged 61.0 ± 7.0, 8 males) with poor response and 15 (aged 62.6 ± 8.3, 5 males) age- (*p* = 0.58), gender-matched (*p* = 0.20) patients responding well, were acquired and analyzed. Multivoxel pattern analysis (MVPA) with a searchlight algorithm and support vector machine (SVM), was applied to identify the spatial pattern of the gray matter (GM) volume, with high predicting accuracy. The predictive regions, with an accuracy higher than 79%, were located within the cerebellum, posterior insular cortex (pIC), middle and orbital frontal lobes (mFC and OFC), anterior and middle cingulum (ACC and MCC), precuneus (PCu) and cuneus. Among these regions, mFC, pIC and MCC displayed significant increases of GM volumes in patients with poor response, compared to those with a good response. The combination of sMRI and MVPA might be a useful tool to explore the neuroanatomical imaging biomarkers of HZ-related pain associated with medication responses.

## Introduction

Resulting from the reactivation of a latent varicella-zoster virus, herpes zoster (HZ) is characterized by a unilateral, localized painful blistering skin rash with the complication of pain varying from burning, tingling, allodynia to hyperalgesia (Johnson and Rice, 2014; Hadley et al., 2016). The primary objectives of treating HZ are to accelerate the healing of skin lesions, and more importantly, to reduce the duration of zoster-associated pain, in order to lower the risk of progression to post-herpetic neuralgia (PHN) (Johnson and Rice, 2014). Medication treatment with central nervous system drugs is commonly used to ease the pain of HZ patients (Harden et al., 2013). Most HZ patients are middle-aged or elderly people with severe pain due to their lower immunity (Jung et al., 2004). They are more likely to suffer from multiple side effects of medication and have poor treatment responses (Jung et al., 2004). However, a great challenge remains for clinicians to evaluate the medication responses, before planning precise treatment protocols, which could further delay the proper treatment exposing patients to the high risk of getting intractable PHN (Hadley et al., 2016). In order to promote the efficiency of medication treatment, as well as to ease the economic and mental burden of HZ patients, it is necessary to explore the neural biomarkers with prognostic value in medication treatment.

Over the last decade, functional MRI (fMRI) has been increasingly applied in studying abnormal brain activity of HZ and PHN patients (Geha et al., 2007, 2008; Liu et al., 2013; Zhang et al., 2014; Jiang et al., 2016; Cao et al., 2017a, 2018; Yu and Yu, 2017; Hong et al., 2018). By contrast, few structural changes of these patients, which potentially underlie the functional abnormalities, have been explored. Recently, a study applying the voxel-based morphometry (VBM) method in structural magnetic resonance imaging (sMRI) (Cao et al., 2018), reported that the development from HZ to PHN was associated with decreased gray matter (GM) volumes in the hippocampus, superior and medial frontal gyrus, thalamus, occipital lobe, and the parietal lobe, as well as increased GM volumes in the bilateral cerebellum, inferior and the middle temporal gyrus. We supposed that the dynamic changes in brain structure not only manifests in the development of HZ to PHN but also occurs at the early initiation of HZ, which may mediate the responses of patients to medication.

Voxel-based morphometry is the most widely used technique to study regional cerebral volume or tissue concentration difference in sMRI analysis (Ashburner and Friston, 2000; Good et al., 2001). As a univariate method, it performs statistical analysis at each voxel separately, either in a ROI-wise or whole brain data-driven manner. There are drawbacks in both methods of analysis. The ROI approach often requires a prior hypothesis of potential pathophysiological brain regions, while whole brain voxel-wise analysis with the restriction of multiple comparisons would cause a loss of sensitivity (Hendler et al., 2014). They are also subjected to a large sample size to attain statistical power. Further, VBM overlooks the dependency of the focal set of voxels in localizing informative regions relevant to anatomical differences of brains (Haynes and Rees, 2006). To overcome these shortcomings, multivoxel pattern analysis (MVPA) has been proposed to take advantage of multiple voxels’ information in depicting the pattern of the human brain (Ecker et al., 2010; Bendfeldt et al., 2012; Haxby, 2012). MVPA has become increasingly popular in neuroimaging research, because it has great benefits, including its efficiency in detecting subtle anatomical differences (Ashburner, 2009; Uddin et al., 2011; Liu et al., 2012; Zhang et al., 2018), and a greater sensitivity and specificity than mass-univariate analyses, with generally complementary results (Haynes and Rees, 2005; Kamitani and Tong, 2005; Jimura and Poldrack, 2012).

Machine learning (ML) based MVPA has been used in previous studies to classify patients from healthy controls (Klöppel et al., 2015; Wolfers et al., 2015; Zhang et al., 2018), or to predict which patients might have different medication responses (Liu et al., 2012; Qin et al., 2015) with high sensitivity and specificity. Of note, the high dimensionality of neuroimaging data is a big challenge when applying ML methods, because voxel-wise features greatly exceed the sample size. In order to achieve a fully data-driven, whole-brain classification based on MVPA, we chose to use the searchlight method combined with the commonly used ML classifier, support vector machine (SVM), in the current study. With each voxel as the center, a searchlight is defined with a spherical set of voxels and then used to train and test SVM. Accuracy of this classifier is assigned to the central voxel of the sphere. Finally, a parametric accuracy map is created and used to identify significant brain regions in classification (Kriegeskorte et al., 2006). The searchlight method is appealing in the following aspects: (a) minimizing the effect of curse of dimensionality (each searchlight includes a few voxels); (b) takes advantage of information from multiple adjacent voxels in pattern detection; (c) produces a whole-brain result map that is superficially similar in appearance to the whole-brain significance maps produced by more familiar mass-univariate analyses (based on the general linear model) (Etzel et al., 2013).

In the present study, we aimed to explore the relationship between potential structural changes and medication responses in HZ patients. We hypothesized that the combination of the sMRI and MVPA technique could detect brain structural differences between HZ patients with different medication responses.

## Materials and Methods

### Subjects

This study was approved by the Ethics Committee of the Shenzhen Sixth Hospital of Guangdong Medical University. In total, 36 subjects were recruited from patients at their first initiation of HZ in this hospital from 1 January 2017 to 30 August 2018. Diagnosis of central neuralgia was performed by experienced clinicians in the Department of Pain Medicine of the Shenzhen Sixth Hospital of Guangdong Medical University, according to general practice guidelines (Jeon, 2015). Pain severity in these patients was evaluated via a 10-point visual analog scale (VAS) every day after hospitalization. Specifically, pain intensity assessed right before MRI scanning and after treatment were termed as pre-scanning VAS and post-treatment VAS, respectively. Since the MRI scan could not be arranged while the patients were hospitalized, MRI data were generally acquired within 3 days after patients were hospitalized, at which time they could already have been medicated or not. For those who were already medicated, two experienced clinicians measured VAS and made sure that effective changes in pain intensity had not occurred before MRI acquisition, which meant that pain intensity in these patients was approximately the same as that before medication. Inclusion criteria were: age between 55 and 79 years old; right-handed; no history of psychiatric or neurological disorders; a primary symptom of HZ-related acute pain (duration of less than 1 month); and a pre-scanning VAS higher than five. Each patient provided written informed consent according to the Declaration of Helsinki.

All patients were administrated according to the standardized medication protocol by experienced physicians for a week. This protocol was normally an individually calibrated with prescriptions of the following systemic central acting agents: the anticonvulsants gabapentin and pregabalin; and/or the tricyclic antidepressants amitriptyline. After a one-week treatment, the HZ patients with a reduction of VAS less than three were defined as having medication-resistant pain (MRP), while others were defined as having medication-sensitive pain (MSP).

Demographic and clinical data were compared between the two groups using two-sample *t*-tests or a Chi-square test in the Statistical Package for Social Science, version 19 (SPSS Inc., United States). The threshold level of significance was set at *p* < 0.05.

### MRI Acquisition

Magnetic resonance imaging scanning was performed on a Siemens Skyra 3.0T scanner with an 8-channel head coil in the Shenzhen Sixth Hospital of Guangdong Medical University. All subjects were instructed to remain still and awake with their eyes closed during scanning. High-resolution T1-weighted structural images were obtained using a Siemens 3D MPRAGE sequence with the following parameters: 320 slices, slice thickness = 0.6 mm, TR/TE = 1900/2.12 ms, field of view = 256 × 256 mm^2^, data matrix = 448 × 448, spatial resolution = 0.57 × 0.57 × 0.60 mm^3^, inversion time = 900 ms, flipped angle = 9°.

### MRI Data Analysis

All sMRI images were visually inspected and those with severe motion artifacts and/or visible anatomical deformation were excluded. We calculated the GM maps using the VBM toolbox^1^ in SPM8 (Version 6313, Wellcome Department of Imaging Neuroscience, London, United Kingdom^2^) imbedded in MATLAB R2013a. The VBM procedure is described briefly as follows: (1) registration to Montreal Neurological Institute (MNI) stereotactic space; (2) segmentation into three types of tissues, namely GM, white matter and cerebrospinal fluid; (3) creation of a study-specific template via the high-dimensional Diffeomorphic Anatomical Registration Through Exponentiated Lie Algebra (DARTEL) algorithm; (4) non-linear registration to the DARTEL existing template; (5) modulation to preserve the total volume of each brain tissue; (6) smoothing using a Gaussian kernel with full-width-half-maximum (FWHM) of 8 mm. Smoothing is a standard step in the VBM analysis pipeline, to render the data more normally distributed and to compensate for the inexact nature of the spatial normalization (Mechelli et al., 2005).

**FIGURE 1:**
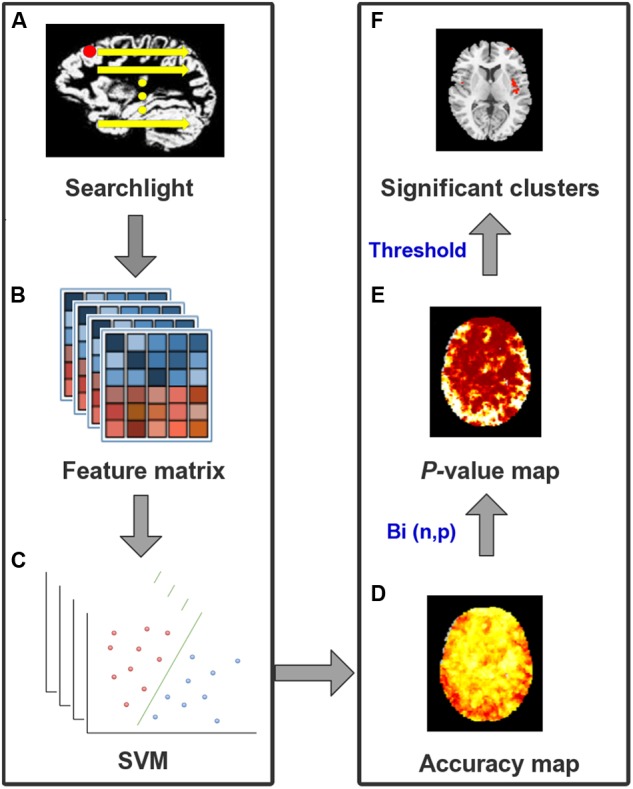
The flowchart for MVPA procedure. **(A)** For each voxel in GM as a center, a 5-mm sphere was defined as a searchlight. **(B)** GM volumes of all voxels in the same sphere were extracted from all subjects to construct a feature matrix. **(C)** SVM classifier with LOOCV was built to produce an accuracy value for the central voxel. **(D)** This procedure was repeated after the whole brain accuracy map was created. **(E)** Binominal distribution, with a null hypothesis that there were no differences between two groups, was tested to convert the accuracy map into a *p-*value map. **(F)** With a threshold of *p* < 0.0001 and cluster size >50 voxels, significant clusters to classify different groups were identified finally.

The MVPA technique implemented in this study a combined searchlight algorithm and support vector machine (SVM) (Uddin et al., 2011). Generally, a searchlight algorithm, which considers the information of multiple voxels, can be more sensitive to group differences over traditional univariate measures (Ecker et al., 2010; Bendfeldt et al., 2012). In our study, MVPA was performed on the smoothed GM maps obtained in the VBM procedure. The flowchart of MVPA based classification is shown in Figure 1 and the details of the MVPA procedure are as follows. First, at each voxel (Vi), a sphere with 5-mm radius was defined centering at Vi. Notice that previous studies defined the radius by experience (Uddin et al., 2011; Liu et al., 2012; Zhang et al., 2018). In our study, we tested different radii (e.g., 8, 10, and 12 mm) and found no significant difference among the ultimate results (Supplementary Figure S1). Besides, a large sphere radius would result in the omission of some subtle spatial pattern information. Thus, we chose a small radius (5 mm) to present the results. First, a high-dimensional feature vector was acquired by extracting the values of all 171 voxels in the sphere. Second, with such a feature vector, a non-linear support vector machine (SVM) with radial basis function (RBF) was applied to predict medication responses using the LIBSVM software^3^. The parameters were set to default to construct the SVM model for each Vi. Then leave-one-out cross-validation (LOOCV) was adopted to yield the classification accuracy of Vi. We split all subjects into a training set (N-1 subjects, N denotes the number of all subjects) and a testing set (the remaining subject). Then two feature matrices M_(N1)^∗^v_ and M_N2^∗^v_ (N1, N2, and V denote the numbers of subjects in the training and testing sets, and the number of voxels in the sphere) representative of the spatial patterns of the two data sets were obtained using the aforementioned high-dimensional feature vectors. Input features of training data were normalized and used to construct an SVM model. After repeating this procedure for all voxels in gray matter, a three-dimensional accuracy map was finally gained to represent the structural pattern of the discriminating ability of classifying MRP from MSP.

To identify clusters with statistical significance, we first converted the accuracy map into a *p*-value map, then conducted a connected algorithm on the *p*-value map to find the clusters that survived the threshold. To be specific, with a null hypothesis that there is no difference between this two groups, we assumed that the accuracy map followed a binomial distribution Bi(n, p) (Pereira et al., 2009). Herein, n denoted the number of all patients and the probability of p was equal to 0.5. When k subjects were successfully classified out of n, we defined the probability of a random variable m exceeding k as a *p*-value. This procedure converts the accuracy map into a *p*-value map. After that, a connected algorithm was applied to the *p*-value map. Compared with previous studies (Uddin et al., 2011; Liu et al., 2012), a stricter threshold for a significant cluster was set as voxel-wise *p* < 0.0001 (corresponding to a voxel-wise accuracy of 79%) with at least 50 adjacent voxels. Therefore, for each voxel with *p* < 0.0001, its 18 neighboring voxels would be examined if their *p*-values below 0.0001. Those satisfied with the threshold would be labeled as a component belonging to the same cluster as the center voxel. After all GM voxels finished labeling, clusters with more than 50 contiguous voxels were extracted as the MVPA clusters.

In order to further evaluate the statistical significance of the detected clusters that survived our threshold, permutation testing with 1000 iterations was implemented (Ojala and Garriga, 2010). The labels (MRP or MSP) were randomly assigned to the input data. LOOCV was used to generate accuracy values for each permutation test. A total of 1000 values were acquired under the null-hypothesis distribution, with which we could calculate the proportion of accuracy values equal to or greater than the real accuracy. When actual accuracy exceeded 95% (namely, one-tailed *p* < 0.05) of resulting values from permutations, it was considered statistically significant.

### *Post hoc* Analyses

To further study the difference of GM volumes between MRP and MSP, *post hoc* analyses in those clusters detected with MVPA were performed. This analysis may also help interpret the potential mechanism underlying drug actions and treatment outcomes. With age and gender as covariates, voxel-wise two-sample *t*-test was performed in these regions to determine the significant differences between MRP patients and MSP patients. AlphaSim correction was performed on the statistical map in the REST software^4^ for multiple comparisons. Specifically, the AlphaSim procedure generates an estimate of the overall significance level achieved from various combinations of the probability threshold and cluster size threshold. Herein, the threshold was estimated to be *p* < 0.01 with a minimum cluster size of 40 contiguous voxels, which was corresponding to *p* < 0.05 before correction.

Additionally, for each subject in the MSP group, the mean GM volume of each identified cluster was calculated. Spearman’s correlation was used to examine the relationship between the mean GM volume and the change of pain intensity after treatment, which was defined as ΔVAS (pre-scanning VAS *minus* post-treatment VAS).

## Results

### Patient Demographics

Three patients were excluded, due to unsatisfactory image quality, and four patients dropped out for alternative therapies. Finally, 29 patients were included for the following data analyses, including 14 MRP and 15 MSP. Demographic and clinical characteristics of the included patients are shown in Table 1. The MRP group (*n* = 14) was comprised of six females and eight males (mean age ± std: 61.0 ± 7.0 years), while the MSP group (*n* = 15) consisted of 10 females and five males (mean age ± std: 62.6 ± 8.3 years). There was no significant difference in age (*p* = 0.58), gender ratio (*p* = 0.20) and pain duration (*p* = 0.07) between the two groups. The Pittsburgh sleep quality index between MSP and MRP showed no significant difference before treatment (Pre_PSQI) and was significantly lower in MSP than in MRP after treatment (Post_PSQI). Between-group comparison of pre-scanning VAS showed an insignificant difference (*p* = 0.09), while post-treatment VAS was significantly higher in MRP patients than in the MSP group (*p* < 0.0001).

**Table 1:** Demographic and clinical characteristics of the MRP group and the MSP group.

Measures	MRP *n* = 14	MSP *n* = 15	*P*-value
Ages	61.0 (7.0)	62.6 (8.3)	0.58^a^
Males/Females	8/6	5/10	0.20^b^
Duration	14.3 (7.6)	9.0 (7.4)	0.07^a^
Pre-VAS	6.9 (1.1)	6.1 (1.4)	0.09^a^
Post-VAS	6.9 (1.5)	2.4 (0.6)	<0.0001^a^
Pre-PSQI	8.9 (3.7)	8.4 (5.7)	0.76^a^
Post-PSQI	7.4 (3.9)	3.7 (1.6)	0.004^a^


*Data are presented as mean (standard deviation). MRP, medication-resistant pain; MSP, medication-sensitive pain; VAS, visual analog scale; Pre-VAS, pre-scanning VAS; Post-VAS, post-treatment VAS; Pre-PSQI, Pittsburgh sleep quality index before treatment; Post-PSQI, Pittsburgh sleep quality index after treatment. ^*a*^two-sample *t*-test; ^*b*^Chi-square test.*

### Spatial Patterns Characterized by MVPA

**FIGURE 2:**
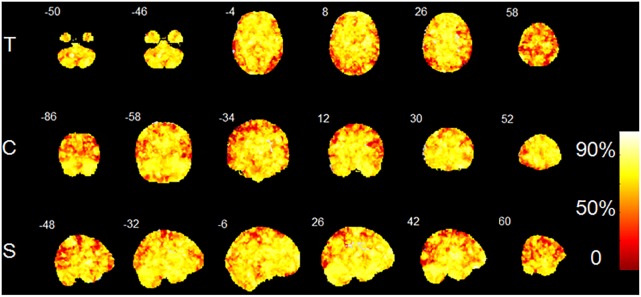
Accuracy map created by MVPA procedure. T, transverse direction; C, coronal direction; S, sagittal direction. The color-bar indicates the classification accuracy values of the whole brain GM voxels. The image is displayed in the neurologic convention, with the left side corresponding to the left-brain hemisphere.

**FIGURE 3:**
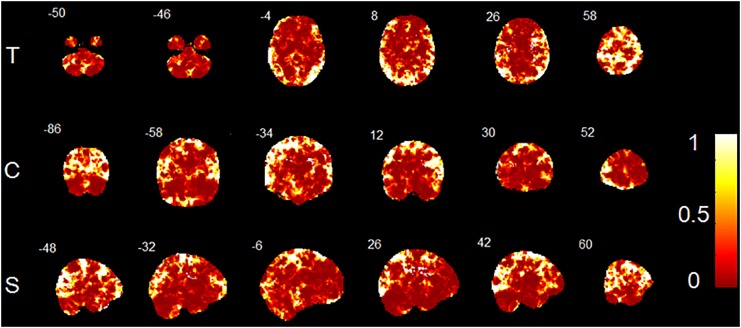
*P*-value map converted from accuracy map. T, transverse direction; C, coronal direction; S, sagittal direction. The color-bar indicates the *p*-values of the whole brain GM voxels. The image is displayed in the neurologic convention, with the left side corresponding to the left-brain hemisphere.

**FIGURE 4:**
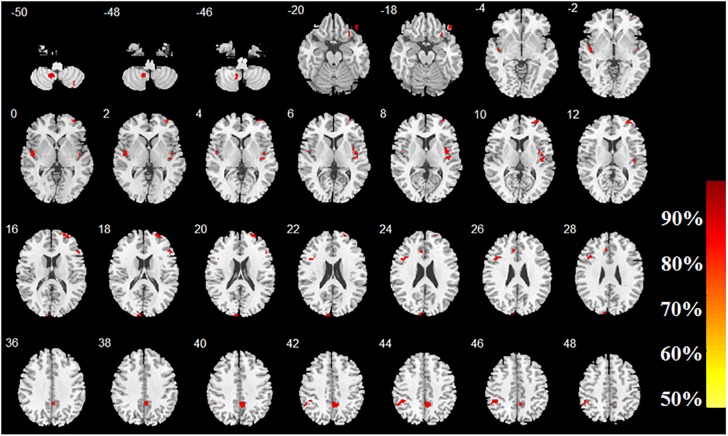
Brain regions with high classification accuracy identified by MVPA. The color bar indicates the classification accuracy of these brain regions. The image is displayed in the neurologic convention, with the left side corresponding to the left-brain hemisphere.

The accuracy map and *p*-value map at the intermediate procedure of MVPA are displayed in Figure 2, 3, respectively. And the spatial patterns of GM maps characterized by MVPA without covariates regression before classifier training are shown in Figure 4. For comparisons between significant clusters detected by MVPA with and without covariates regression, please see Supplementary Figure S2. The clusters included in the spatial patterns have a voxel-wise accuracy of at least 79% for the classification between MRP and MSP. The peak accuracy values of these clusters are reported in Table 2. The prominently discriminative cortical and subcortical areas included bilateral posterior cerebellum, bilateral superior temporal lobe mostly extending to the posterior insular cortex (pIC), inferior orbital frontal cortex (OFC, right), middle frontal cortex (mFC, right), inferior frontal lobe (IFC, bilateral), anterior and middle cingulum (ACC and MCC), inferior parietal lobe (IPL), precuneus (PCu) and the cuneus. All clusters detected by MVPA exhibited a statistical significance in the permutation test (*p* < 0.05, Table 2).

**Table 2:** Brain regions with high predictive accuracy identified by MVPA.

Brain regions (AAL)	Cluster Size (voxels)	Peak MNI coordinates			Peak Acc (%)	*P*-value
		**X**	**Y**	**Z**		
Cerebelum_8_R	90	35	-66	-51	83	0.003
Cerebelum_9_L	171	-11	-56	-50	90	0.003
Frontal_Inf_Orb_R	95	29	27	-23	83	0.001
Frontal_Inf_Orb_R	60	45	39	-20	90	0.003
Frontal_Inf_Tri_R	57	50	27	18	86	0.004
Frontal_Inf_Tri_L	144	-44	14	26	90	0.003
Temporal_Sup_L	194	-47	-14	-3	86	0.002
Temporal_Sup_R	181	42	-18	2	86	0.001
Insula_R	182	45	-9	-2	79	0.001
Frontal_Mid_R	352	33	56	0	86	0.004
Cuneus_L	128	-8	-98	15	86	0.001
Cingulum_Mid_R	303	3	-48	36	83	0.003
Cingulum_Ant_L	62	-8	26	24	79	0.004
Parietal_Inf_L	195	-47	-41	44	90	0.002


*AAL, anatomical automatic labeling; MNI, montreal neurological institute; L, left hemisphere; R, right hemisphere; Acc, accuracy. The brain regions contain the voxels with an accuracy of at least 79%. The maximum accuracy value among voxels in each cluster was obtained to be the peak accuracy for that cluster. The *p*-values were obtained by permutation tests with 1000 iterations.*

### *Post hoc* Analyses

**FIGURE 5:**
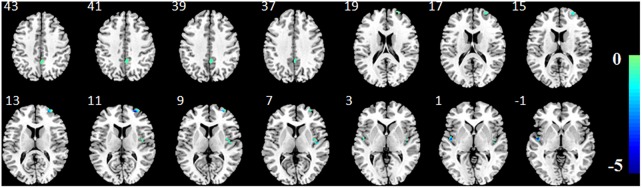
Brain regions with significant GM volume differences between MRP patients and MSP patients by *post hoc* VBM analysis. The color bar shows the corresponding peak *t*-values of the clusters and the negative values imply smaller GM volumes in patients with MRP than those with MSP. The image is displayed in the neurologic convention, with the left side corresponding to the left-brain hemisphere.

Two-sample *t*-tests with an AlphaSim correction (*p* < 0.05, cluster size >40) showed that five out of the 14 clusters identified by MVPA, exhibited significant GM volume decreases in MRP patients, compared to those with MSP (Table 3 and Figure 5). These brain regions consisted of the frontal lobes (mFC and OFC, right), superior temporal lobes (mainly pIC, bilateral), and MCC (extending to PCu). No regions showed significantly higher GM volumes in MRP patients compared to the MSP patients.

**Table 3:** Brain regions with GM volume differences between MRP patients and MSP patients by *post hoc* VBM analysis.

Brain regions (AAL)	Cluster size (voxels)	Peak MNI coordinates			Peak *t*-value
			
		X	Y	Z	
MRP<MSP					
Frontal_Mid_R	273	27	54	11	-4.67
Temporal_Sup_L	100	-48	-9	0	-4.81
Temporal_Sup_R	49	45	-14	6	-4.21
Insula_R	98	39	-9	6	-3.87
Cingulum_Mid_R	268	5	-47	39	-3.42


*AAL, anatomical automatic labeling; MNI, montreal neurological institute; L, left hemisphere; R, right hemisphere; MRP, medication-resistant pain; MSP, medication-sensitive pain. The significance level was set at *p* < 0.05 and cluster size >40 voxels (AlphaSim corrected).*

In the MSP group, no significantly positive or negative correlations were detected between GM volumes in MVPA-identified regions and ΔVAS.

## Discussion

In this study, we applied MVPA to characterize the potential neurological biomarkers in sMRI to predict the medication responses of HZ patients. The cerebellum, insula, frontal lobe, ACC and PCu showed a pattern of high classification accuracy using the MVPA method. These regions implied a deficiency in both the sensory-discriminative and affective/cognitive aspects of pain processing in HZ patients, as shown in previous studies based on brain MRI (Yu and Yu, 2017; Cao et al., 2018; Hong et al., 2018).

Antidepressants and anticonvulsants are uniformly recommended as first-line medications for neuropathic pain caused by herpetic infections (Gore et al., 2007). In the current study, a combination of these two kinds of drugs were prescribed to HZ patients for pain management. The pharmacological mechanisms of anticonvulsants and antidepressants are different. Anticonvulsants, such as gabapentin and pregabalin, bind to the alpha-2-delta protein to reduce the release of excitatory neurotransmitters like glutamine and noradrenaline (Gore et al., 2007). And tricyclic antidepressants, amitriptyline, inhibits the reuptake of serotonin and noradrenaline and increases their concentration in intrasynaptic space (Coluzzi and Mattia, 2005), which could modulate activity in endogenous descending pain inhibitory pathways. Distinct actions of antidepressants and anticonvulsants on the neurotransmitter system could both produce antinociception, analgesic and anxiolytic activity.

However, the central mechanisms of these drugs for pain relief are largely unclear. Though limited, there are some studies based on MRI techniques which reveals that activity or morphology changes in frontal lobe, insular cortex, ACC and cerebellum of neuropathic pain were affected by these central nervous system drugs (Marchand, 2008). Structural/functional deficiency in these regions was not only detected in our study, but also in other previous studies on neuropathic pain, such as chronic lower back pain (Buckalew et al., 2008), headache (Dettmers et al., 2001; Naegel et al., 2014) and fibromyalgia (Shi et al., 2016). Therefore, antidepressants and anticonvulsants may have potential effects on these brain regions of HZ patients.

Posterior insular cortex receives nociceptive signals from the thalamus-spinal ascending system (Davis and Moayedi, 2013) because it has direct anatomical connections to spinothalamic tracts. Gabapentin could reduce the activations in the bilateral operculoinsular cortex to modulate nociceptive transmission in humans (Iannetti et al., 2005). Additionally, for individuals with evoked or clinical pain, the concentration of glutamine in pIC changed after non-pharmacological treatment (Harris et al., 2008). A study on fibromyalgia patients (Harris et al., 2013) also found decreased glutamine in this region after pregabalin treatment rather than a placebo, suggesting that the insular cortex could be a potential target for pregabalin. Our findings showed significant decrease of GM in bilateral pIC of MRP patients compared to MSP patients. Since inhibition of neural activity and neurotransmitter (e.g., glutamine) release in pIC are closely related to pain remission, structural deficiency in this region may have a negative impact on its activity modulated by drugs, thus resulting in the poor response of MRP patients.

The cingulate cortex and prefrontal lobe are critical regions for emotional and cognitive regulation of perceived pain. Accumulating evidence from fMRI studies showed an abnormal cingulum and frontal lobe response in HZ and PHN patients compared to healthy controls (Peyron et al., 2000; Geha et al., 2007, 2008; Liu et al., 2013; Dong et al., 2014, 2015; Jiang et al., 2016; Cao et al., 2017b; Hong et al., 2018), suggesting that dysfunction in controlling emotion, anticipation and evaluation could modify pain perception. In line with these studies, in our study ACC and PFC structural impairments were observed in HZ patients, supported by a significantly increased GM volume of the cingulate cortex in MRP rather than MSP. It may indicate that ACC and PFC could be important regions that influence pain regulation in patients with HZ-related pain or PHN. These two regions could even be potential targets of neuropathic pain drugs, since accumulating evidence has proven the regulation of activity by antidepressants and anticonvulsants in ACC and PFC. In mice with neuropathic pain induced by partial sciatic nerve ligation (Wang et al., 2015), pregabalin treatment decreased the expression of c-Fos, an indicator of transient and rapid neuronal activity, in neurons of the ACC. Another study (Lin et al., 2014) reported that synaptic transmission of ACC in adult mice could be inhibited by gabapentin. For healthy volunteers with capsaicin-induced central sensitization (Iannetti et al., 2005), gabapentin also suppressed stimulus evoked deactivation in PFC. When irritable bowel syndrome patients were stressful (Morgan et al., 2005), their pain related activation of ACC and left posterior parietal cortex were reduced by amitriptyline compared with baseline state. Altogether, we speculate that compared to MSP, MRP patients with more negative anatomy changes in the cingulate cortex and PFC, may respond insufficiently to medication, which may result in lessened remission of pain after treatment.

Patients with neuropathic pain are often characterized by symptoms of mood disorders (Inoue et al., 2017). This comorbidity supports that neuropathic pain and affective disturbances may share some common pathogenetic mechanisms (Aloisi et al., 2016). HZ patients in our study also manifested psychological distress and loss of sleep due to severe pain, supported by relative higher PSQI scores compared to healthy people before treatment. After treatment with antidepressants and anticonvulsants, MSP patients received a mood boost and their sleep recovered. Medial PFC is one of the most common brain regions involved with pain in affective disturbances (Descalzi et al., 2017). Not surprisingly, we also found a PFC morphologic difference between MSP and MRP patients, which could predict pharmacological responses accurately. Experiments from preclinical models demonstrated that neuroinflammation in affective forebrain regions would be evoked by nerve injury, and disrupts the normal physiological process which deals with affective disturbances (Fiore and Austin, 2016). It turns out that neurogenesis in adults can be modulated by psychoactive agents, including antidepressants and anticonvulsants (Fiore and Austin, 2016). Therefore, these drugs may help to eliminate inflammation, promote neuronal growth in affective brain regions, and result in an analgesic effect in HZ patients. Since MRP patients suffered prolonged structural changes in PFC compared to MSP patients, they may not benefit from drugs for such a short duration of treatment, which needs to be confirmed by a longitudinal follow-up study.

PFC, IPL, and PCu are hub regions included in the default mode network (DMN), which is often deactivated during painful stimuli and other tasks (Iannetti et al., 2005). We found that structural differences in these DMN components were able to predict medication response in HZ patients. An fMRI study demonstrated that gabapentin had an antihyperalgesic effect by reducing the magnitude of deactivation in DMN regions in central sensitization, but not in a normal state (Iannetti et al., 2005). Evidence from an EEG study stated that PCu activation was correlated with pain sensitivity (Goffaux et al., 2014). It is possible that antidepressants could modulate DMN activity of HZ patients to restore their function from central sensitization, such as hyperalgesia and allodynia which commonly occurs in neuropathic pain patients. Additionally, genetic and environmental factors that contribute to pre-existing structural differences in DMN regions, would be partly related to the medication response of HZ patients. This could be further supported by a sMRI study, which found that the higher the pain sensitivity in healthy individuals, the less GM volume was present in their PCu (Emerson et al., 2014). This finding may suggest that a poor response to medication for MRP patients, could possibly be due a significantly lower GM volume in DMN regions compared to that in MSP patients. Though investigations regarding the effects of antidepressants and anticonvulsants on neural plasticity remains lacking, such kind of structural deficiency might not be easily changed by medication.

The posterior lobes of the cerebellum in our study also yielded high classification accuracy. Recently, a multimodality MRI study described GM volume increase in posterior cerebellum during the transition from HZ to PHN (Cao et al., 2018). Thus, the cerebellum may play an important role in the development of chronic pain. So far, no studies have reported any direct response of cerebellum to antidepressants or anticonvulsants in neuropathic pain. But noradrenergic and serotoninergic systems, originating in the brain stem and projecting to the spinal cord dorsal horn, could be influenced by antidepressants, leading to the modulation of pain perception (Valverderk et al., 1994). Since the brain stem has a direct connection to the cerebellum, it cannot be excluded that the activity of the cerebellum would be indirectly affected by antidepressants through the changes of neurotransmitter levels in the brain stem. This indicates that a difference in the cerebellum between MSP and MRP patients, may potentially mediate the different responses to medication.

Several brain regions including the OFC, ACC, PCu and the cuneus showed a significant predictive power to classify MRP from MSP patients. However, GM volumes of these areas did not show significant differences between MSP and MRP in our *post hoc* VBM analysis. This observation may be mainly attributed to the methodological difference between the two methods. Specifically, VBM applies voxel-wise two-sample *t*-test between groups, while MVPA extracts values of all voxels in the sphere as informative features and takes full advantage of the machine learning algorithm to eventually learn good feature representations for classification. Therefore, MVPA would be more sensitive in detecting subtle differences in the aforementioned brain regions between the two groups, as compared to VBM. In this view, the performance of MVPA may be better than the traditional univariate VBM method.

Some limitations of the present study need to be considered. First, the sample size is relatively small, even though it seems moderate compared to previous studies that had sample sizes varying from 11 to 22 to explore HZ- or PHN-related brain abnormality (Geha et al., 2007, 2008; Liu et al., 2013; Zhang et al., 2014, 2016; Jiang et al., 2016; Cao et al., 2017a,b, 2018). We performed a *post hoc* estimation of the sample size required for detecting the difference of GM volumes in the brain regions characterized by MVPA. Average GM volumes of MVPA-detected clusters were used to calculate the possible effect sizes, because GM volume was the variable required to distinguish MSP from MRP. With such effect sizes we calculated the sample size to be around 8∼25 patients per group. This may partly justify that the sample size in the current study could be appropriate in yielding a reliable result. It should, however, be noted that future work, with a larger sample, especially from a multicenter, would be necessary to further verify these preliminary findings. Second, an Alphasim correction using the Monte Carlo simulation was applied to control type I error in *post hoc* VBM analysis, which was insufficient compared to the standard FWE or FDR correction. We have conducted FWE and FDR correction on VBM statistical maps but found no surviving clusters. This is partly due to a small sample size in our study. Previous studies have justified that a sample size smaller than 40 in each group, would reduce the reproducibility of results variously, no matter which multiple comparisons strategy was adopted (Button et al., 2013; Chen et al., 2018). Though less strict than FDR and FWE corrections, Alphasim correction is commonly used in the neuroimaging field with a reasonable underlying principle that “true regions of abnormality will tend to occur over contiguous voxels, whereas noise has much less of a tendency to form clusters of distinguish between signal and noise” (Ward, 2000). As an exploratory study, the present work adopted the Alphasim correction to provide some illuminating results to deepen our understanding of potential drug actions on the central system of HZ patients. Therefore, further studies with a strict multiple comparisons approach are needed to confirm these preliminary results. Third, when using MVPA, it is still challenging to interpret the inherent nature of the structural pattern that leads to an accurate prediction. One possible scheme would be longitudinal studies, which could assist in monitoring the dynamic changes of the brain structure. Third, the treatment effect of HZ patients with polypharmacy is often superior to that of monotherapy. However, medication actions are far more complex in polypharmacy treatment. Thus, findings from the current study may not be sufficient enough to elucidate whether synergy or single actions of these drugs mediate the response of brain regions of HZ patients. To explore a specific drug effect on the central nervous system, monotherapy studies are warranted in the future. Finally, a collective dataset of different modalities, such as functional MRI and DTI, would be helpful to depict explicit neural intersections of spatially distributed brain areas.

## Conclusion

In this study, MVPA was applied to a structural MRI to characterize the spatial patterns in predicting the medication responses of HZ patients. The anatomical deficiency in MRP and MSP patients was mainly identified in the insula, ACC, MCC, PFC, IPL, PCu, cuneus and the cerebellum, which are all highly involved in the sensory, emotional and cognitive aspects of pain. These findings may provide new insights into the neural biomarkers that could serve as medication targets for HZ-related pain. Such insights could also assist in providing precise clinical interventions to expedite patient recovery and to prevent the progression to intractable PHN.

## Ethics Statements

The protocol was approved by the ethics committee of the Shenzhen Sixth Hospital of Guangdong Medical University. All subjects provided written informed consent in accordance with the Declaration of Helsinki.

## Author Contributions

JH, CQ, and YL collected the data. PZ and SW analyzed the data. FC, WS, WT, JZ, ZY, SZ, ZZ, LX, and BH discussed the results.

## Conflict of Interest Statement

The authors declare that the research was conducted in the absence of any commercial or financial relationships that could be construed as a potential conflict of interest.

## Footnotes

1 http://dbm.neuro.uni-jena.de/vbm8/

2 http://www.fil.ion.ucl.ac.uk/spm

3 https://www.csie.ntu.edu.tw/∼cjlin/libsvm/

4 http://sourceforge.net/projects/resting-fmri
