# Supplementary material for: Characterizing the Structural Pattern Predicting Medication Response in Herpes Zoster Patients Using Multivoxel Pattern Analysis
Source: Front Neurosci. 2019 May 28;13:534. doi: 10.3389/fnins.2019.00534 (PMC6546876; doi:10.3389/fnins.2019.00534)
Supplement: Supplementary file 1 [file Presentation_1.pdf]

Supplementary Figures

Fig. S1. *Significant clusters detected by MVPA with different radii of searchlight sphere.*

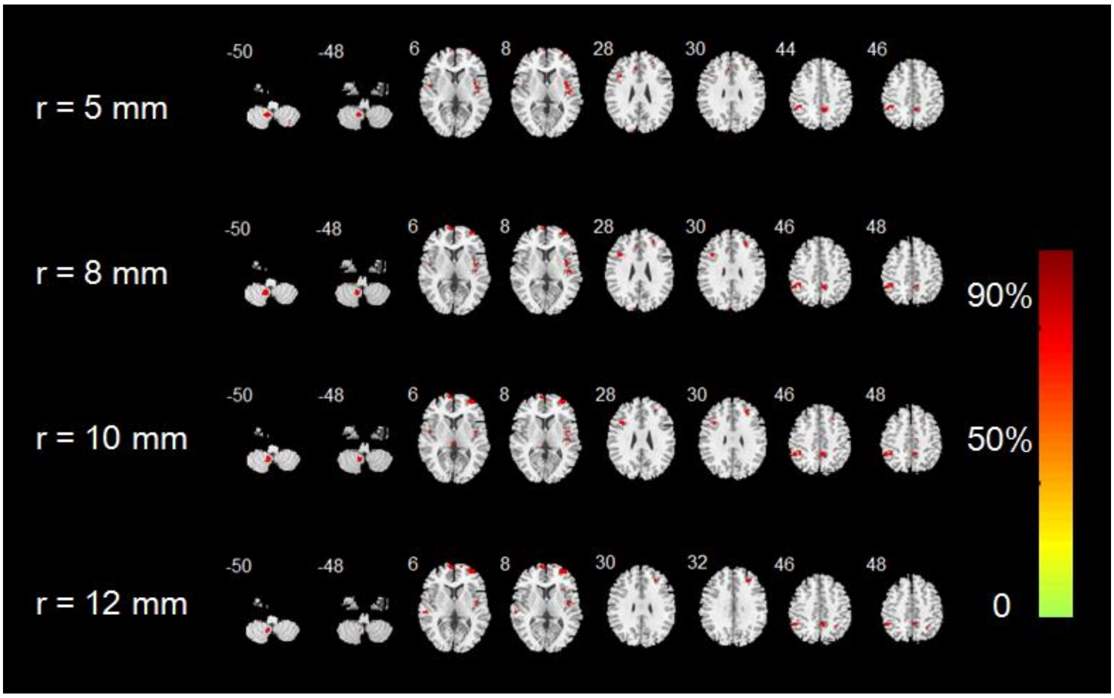

The color bar indicates the classification accuracy of these brain regions. These images are displayed in the neurologic convention, with left side corresponding to left brain hemisphere.

Fig. S2. *Significant clusters detected by MVPA with and without covariates regression in data preprocessing.*

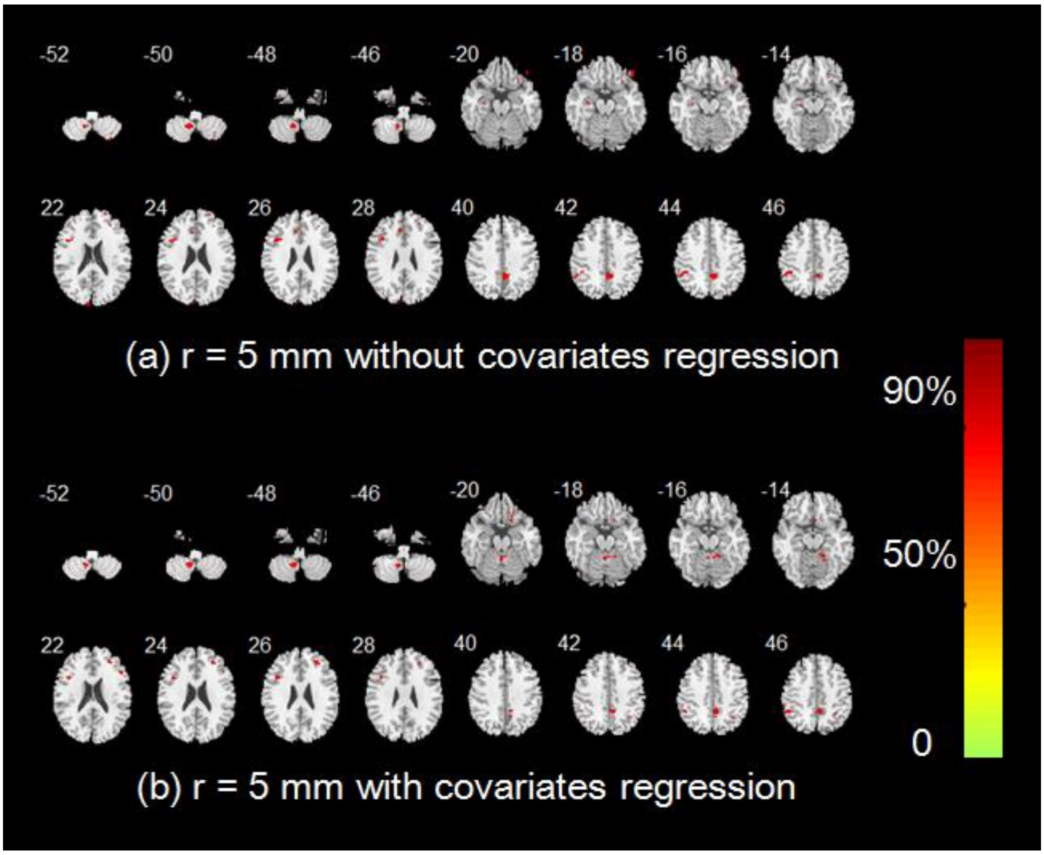

MVPA was conducted on a searchlight sphere with 5 mm radius. (a) Without age and gender regressed in data preprocessing; (b) With age and gender regressed in data preprocessing. MVPA identified significant clusters to classify MRP from MSP ($p<0.0001$, cluster size > 50, corresponding to a voxel-wise accuracy above 79%). The colorbar indicates the classification accuracy of these brain regions. These images are displayed in the neurologic convention, with the left side corresponding to the left brain hemisphere.
